# Efficacy of human coronavirus immune convalescent plasma for the treatment of corona virus disease -19 disease in hospitalized children: A protocol for systematic review and meta analysis

**DOI:** 10.1097/MD.0000000000022017

**Published:** 2020-11-06

**Authors:** Hua Bai, Yongjia Ji, Jia Wang, Xuehong Zhang

**Affiliations:** a General Hospital of Ningxia Medical University; b Ningxia Maternal and Child Health Care Hospital (Ningxia Children's Hospital), Ningxia; c Huazhong university of science and technology union shenzhen hospital, Shenzhen, China.

**Keywords:** children, convalescent plasma, COVID-19, meta analysis, protocol, systematic review

## Abstract

**Background:**

Severe acute respiratory syndrome coronavirus 2 viral infection resulting in corona virus disease 2019 (COVID-19) disease has recently been designated by the World Health Organization as a global pandemic. Some doctors are using convalescent plasma (CP) therapies to treat COVID-19 patients. However, whether CP therapy is effective for children with COVID-19 remains controversial. Therefore, this study further explores the effectiveness and safety of human coronavirus immune CP in the treatment of COVID-19 in children.

**Methods:**

Comprehensively search the electronic databases such as the Cochrane Library, PubMed, EMBASE, Web of Science, China National Knowledge Infrastructure, and WanFang, and collect relevant documents. We will also look for other sources. All document sources will not be restricted by language and publication status. Two researchers will independently conduct research selection, data extraction and research quality assessment. RevMan 5.3 was used for statistical analysis.

**Results:**

This study will provide high-quality comprehensive evidence for the effectiveness and safety of human coronavirus immuno CP in the treatment of COVID-19 in children

**Conclusions:**

The results of this study will provide the basis for the effectiveness and safety of human coronavirus immuno CP treatment of COVID-19 in children.

**PROSPERO Registration number:**

CRD42020199410

## 1 Introduction

Coronavirus disease 2019 (COVID-19) refers to pneumonia caused by severe acute respiratory syndrome (SARS) coronavirus 2 infection.^[1]^ SARS coronavirus 2 is highly infectious and poses a great challenge to national public health.^[2–6]^ The main manifestations of COVID-19 patients are fever, fatigue, and dry cough. Upper respiratory symptoms such as nasal congestion and runny nose are rare, and hypoxia may occur. About half of the patients developed dyspnea after 1 week. In severe cases, they rapidly progressed to acute respiratory distress syndrome, septic shock, difficult to correct metabolic acidosis, bleeding and/or coagulopathy, and a few patients were critically ill and even died.^[7]^ Currently, there is no specific treatment for COVID-19, and isolation treatment and symptomatic supportive treatment are the main ones.

Convalescent plasma (CP) therapy is a form of passive immunity in which antibody-rich blood is collected from recovered patients, processed and then transfused into other patients.^[8]^ The neutralizing antibody is a key influencing factor: it binds to the virus, blocks the virus from entering the cell, regulates the immune system, mediates the phagocytosis of immune cells, and eliminates the virus.^[9]^ CP is used to treat Ebola, SARS, middle east respiratory syndrome coronavirus, pandemic influenza and other unexpected major infectious diseases.^[8]^ In addition, related research has also made some progress.^[10–12]^ The Food and Drug Administration has approved the use of CP to treat patients with severe new coronary pneumonia.^[13]^

However, there is still a lack of evidence regarding the treatment of COVID-19 in children with human coronavirus immune CP therapy. Therefore, this article will evaluate whether the human coronavirus immune CP therapy is effective and safe to treat children with COVID-19. This meta-analysis will be the first study to evaluate whether human coronavirus immune CP therapy is effective and safe for children with COVID-19.

## 2 Methods

### 2.1 Study registration

This study was registered through PROSPERO (PROSPERO Registration number: CRD42020199410). We organize this study based on the Preferred Reporting Items for Systematic Reviews and Meta-Analyses Protocols guidelines.^[14]^

### 2.2 Ethics

Ethical approval is not required as there is no patient recruitment and personal informationcollection, and the data included in our study are from published literature.

### 2.3 Inclusion criteria for study selection

#### 2.3.1 Types of studies

We only collect randomized controlled trials (RCTs) of human coronavirus immune recovery period plasma in the treatment of children with COVID-19. Regardless of language and publication status restrictions.

#### 2.3.2 Types of participants

Inclusion Criteria:

(1) Age 0 to < 18years old;
(2) Hospitalized with symptoms compatible with COVID-19 illness;
(3) Laboratory-confirmed SARS-CoV-2 infection as determined by Polymerase Chain Reaction, or other commercial or public health assay
(4) ABO compatible CP available

Exclusion Criteria:

(1) Onset of symptoms began >12 days before screening;
(2) History of adverse reactions to blood products or other contraindication to transfusion;
(3) Refusal of plasma for religious or other reasons;
(4) Acute heart failure with fluid overload;
(5) Any condition or diagnosis, that could in the opinion of the Site Principal Investigator interfere with the participant's ability to comply with study instructions, or put the participant at risk;
(6) Anticipated discharge within 24 hours

#### 2.3.3 Types of intervention

##### 2.3.3.1 Experimental interventions

Participants will receive COVID-19 CP plus standard of care while being hospitalized for COVID-19.

##### 2.3.3.2 Control interventions

Participants will receive standard of care while being hospitalized for COVID-19.

#### 2.3.4 Types of outcome measures

##### 2.3.4.1 Primary outcome

(1) Clinical recovery (time frame: at day 30), defined in the last 24 hours as normal respiratory and heart rate (or return to baseline, absence of fever, absence of low blood pressure, oxygen saturation greater than 94% or room air (or return to baseline), no need for intravenous fluids (or return to baseline).
(2) SARS-CoV-2 DNA.
(3) SARS-CoV-2 antibody levels.

##### 2.3.4.2 Secondary outcomes

(1) 28-day mortality rate.
(2) C-reactive protein.
(3) Interleukin- 6.
(4) Lactate dehydrogenase.
(5) Creatine kinase.
(6) Liver function.
(7) Runal function.

### 2.4 Exclusion criteria

(1) Incomplete data or misrepresentation of data reports.
(2) Repeated publication of documents.
(3) Case reports, reviews, and so on.
(4) Inability to obtain original documents.

### 2.5 Search methods for identification of studies

A comprehensive search of electronic databases such as The Cochrane Library, PubMed, EMBASE, Web of Science, China National Knowledge Infrastructure, and WanFang, to collect RCTs of human coronavirus immuno CP treatment of children with COVID-19. We will also look for other sources. All document sources will not be restricted by language and publication status. The search strategy performed for the PubMed is shown in Table 1. Searches of other databases are also performed according to this search strategy.

**Table 1:** PubMed search strategy.

Number	Search terms
1	Corona Virus Disease 2019 [Title/Abstract]
2	COVID-19 [Title/Abstract]
3	novel coronavirus[Title/Abstract]
4	Novel coronavirus pneumonia[Title/Abstract]
5	#1 OR #2 OR #3 OR #4
6	convalescent plasma [Title/Abstract]
7	COVID-19 serotherapy [Title/Abstract]
8	COVID19 serum therapy [Title/Abstract]
9	coronavirus disease-19 serotherapy[Title/Abstract]
10	CP [Title/Abstract]
11	coronavirus disease 2019 serotherapy [Title/Abstract]
12	COVID-19 serum therapy [Title/Abstract]
13	COVID-19 convalescent serum treatment [Title/Abstract]
14	SARS-CoV-2 convalescent sera treatment [Title/Abstract]
15	SARS-CoV-2 convalescent serum treatment[Title/Abstract]
16	Covid-19 convalescent sera treatment [Title/Abstract]
17	convalescent serum treatment for Covid-19 [Title/Abstract]
18	COVID-19 hyperimmune globulin therapy[Title/Abstract]
19	COVID19 hyperimmune globulin therapy[Title/Abstract]
20	hyperimmune globulin therapy for COVID-19 [Title/Abstract]
21	COVID-19 convalescent plasma treatment [Title/Abstract]
22	convalescent plasma treatment for Covid-19 [Title/Abstract]
23	SARS-CoV-2 convalescent plasma treatment [Title/Abstract]
24	#6 OR #7 OR #8 OR #9 OR #10 OR #11 OR #12 OR #13 OR #14 OR #15 OR #16 OR #17 OR #18 OR #19 OR #20 OR #21 OR #22 OR #23
25	Children[Title/Abstract]
26	Child[Title/Abstract]
27	#25 OR #26
28	#5 AND #24 AND #27

### 2.6 Data collection and analysis

#### 2.6.1 Data extraction and management

**Figure 1:**
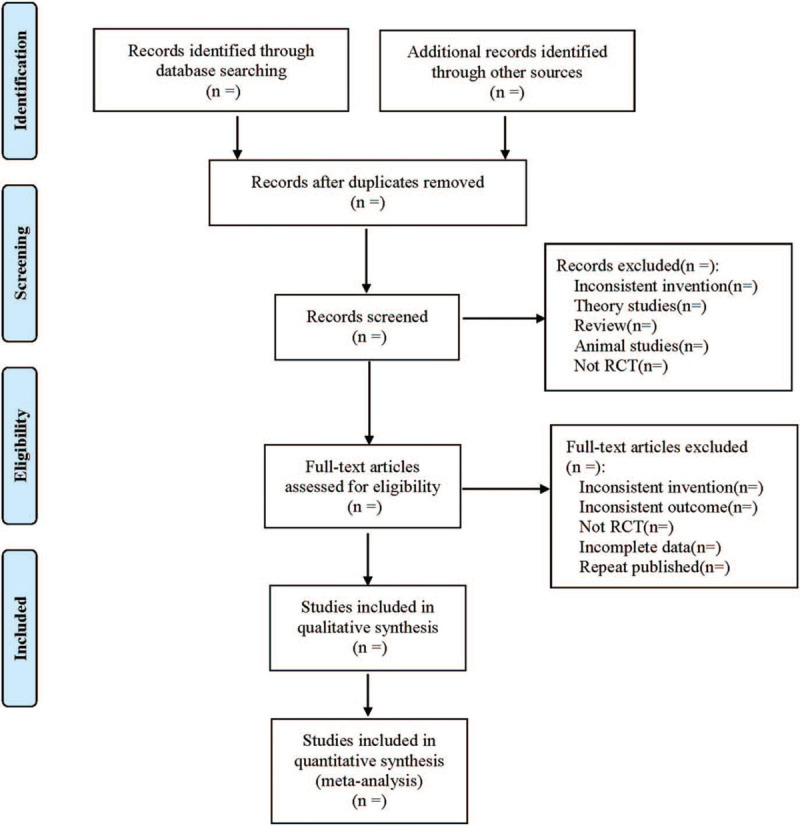
Flow diagram.

Two reviewers independently screened the literature, extracted data, and cross-checked. In case of disagreement, consult a third party to assist in the judgment, and try to contact the author to supplement the lack of data. When selecting documents, first read the title and abstract, and after excluding obviously irrelevant documents, read the full text further to determine whether it will be included. The data extraction content mainly includes: ① Basic information of the included literature, including the first author, country, year of publication, etc; ② Specific details of intervention measures, including the usage of rehabilitation plasma, dosage treatment, and so on; ③ Baseline characteristics of the included subjects; ④ Risk of bias The key elements of evaluation; ⑤ The outcome indicators and outcome measurement data concerned, such as Clinical recovery, SARS-CoV-2 DNA, SARS-CoV-2 antibody levels, so on. The literature screening process is shown in Figure 1.

#### 2.6.2 Assessment of risk of bias

Two researchers used the RCTs bias risk evaluation tool in Cochrane System Review Manual 5.1.0 to evaluate the bias risk of the included RCTs and cross-check the results.

#### 2.6.3 Measures of treatment effect

Count data uses relative risk as the effect indicator, and measurement data uses the standard mean difference as the effect indicator. Each effect size is given its point estimate and 95% confidence intervals.

#### 2.6.4 Dealing with missing data

If the relevant data in the literature is incomplete, the first author or corresponding author will be contacted by email or phone to obtain the missing data. If the missing data is still not obtained through the above methods, we can synthesize the available data in the preliminary analysis. In addition, sensitivity analysis will be used to assess the potential impact of missing data on the overall results of the study.

#### 2.6.5 Assessment of heterogeneity

The 2 researchers used the *χ*2 test to analyze the heterogeneity between the results of the included studies (test level is α = 0.1) and combined with *I*^*2*^ to quantitatively judge the heterogeneity. *I*^*2*^ < 50%, *P* > 0.1 means reasonable heterogeneity, and a fixed-effects model will be conducted. *I*^*2*^ ≥ 50%, *P* < .1 exerts significant heterogeneity, and a random effects model will be conducted.

#### 2.6.6 Assessment of reporting biases

If there are no less than 10 articles included, we will use a funnel chart to test publication bias.^[15]^

#### 2.6.7 Data synthesis

RevMan 5.3 software for statistical analysis. If the statistical heterogeneity between the results of each study does not exist or is small (*I*^2^ < 50%, *P* > .1), the fixed-effects model is used for Meta-analysis; if there is large statistical heterogeneity between the results of the studies (*I*^*2*^ ≥ 50%, *P* < .1), then further analyze the source of heterogeneity. After excluding the influence of obvious clinical heterogeneity, the random effects model is used for Meta-analysis. The level of Meta-analysis is set to α = 0.05. Obvious clinical heterogeneity is treated by subgroup analysis or sensitivity analysis, or only descriptive analysis.

#### 2.6.8 Subgroup analysis

We will conduct subgroup analysis based on different reasons. Heterogeneity is mainly manifested in several aspects such as race, age, condition, dosage of medication, and duration of treatment.

#### 2.6.9 Sensitivity analysis

The robustness of the research results is tested by excluding low-quality studies and conducting sensitivity analysis.

## 3 Discussion

CP is a plasma preparation obtained by a patient who stimulates humoral immunity to produce specific antibodies against the pathogen during a pre-infection and collects the patient's plasma after the patient recovers.^[16]^ In patients with severe infection, infusion of CP neutralizes the pathogens in the body, removes pathogens in the blood circulation, and combines with other antiviral drugs and supportive treatments to achieve the effect of relieving the condition. Studies have also shown that this passive immunity may also be suitable for tumor treatment.^[17]^ When infectious diseases are prevalent, CP may be an effective treatment option in the absence of specific drugs and vaccines. This treatment technology has been used in previous large-scale viral epidemics and has saved many acute infectious diseases, such as middle east respiratory syndrome coronavirus, avian influenza (H1N1, H5N1 and H7N9, etc.), SARS, ebola virus, and so on.^[18–22]^ With the spread of COVID-19, for severe and critical cases, in addition to symptomatic treatment, respiratory support, and circulatory support, CP treatment is also an important treatment measure to save patients.^[23–26]^ This plan will evaluate whether the human coronavirus immune CP is effective and safe for the treatment of COVID-19 in children and provide more accurate and objective evidence for the clinic.

However, our meta-analysis may have some limitations. Both Chinese and English research forms may increase the bias of research. Secondly, the diversity of race, age, drug dosage, and treatment course resulted in higher clinical and statistical heterogeneity.

In summary, this study will help determine the effectiveness and safety of CP therapy for children with COVID-19. We hope that this study can provide higher-quality evidence for the effectiveness and safety of CP therapy in the treatment of COVID-19 in children.

## Author contributions

**Data collection:** Hua Bai and Xuehong Zhang.

**Funding acquisition:** Xuehong Zhang.

**Resources:** Yongjia Ji and Jia Wang

**Software:** Yongjia Ji and Jia Wang.

**Supervision:** Yongjia Ji and Jia Wang.

**Writing – original draft:** Hua Bai and Xuehong Zhang.

**Writing – review & editing:** Hua Bai and Xuehong Zhang.
